# Nutritional Status of Children Aged 12 to 36 Months in a Rural District of Hungyen Province, Vietnam

**DOI:** 10.1155/2019/6293184

**Published:** 2019-04-11

**Authors:** Dang Van Chuc, Nguyen Xuan Hung, Vuong Thi Trang, Dang Viet Linh, Pham Minh Khue

**Affiliations:** ^1^Haiphong University of Medicine and Pharmacy, Haiphong, Vietnam; ^2^Hungyen Obstetrics and Pediatrics Hospital, Hungyen Province, Vietnam; ^3^Haiphong Children Hospital, Haiphong, Vietnam

## Abstract

**Objective:**

To evaluate the nutritional status of children from 12 to 36 months of age in Kimdong, a rural district in Hungyen Province, Northern Vietnam, in 2017.

**Subjects and Methods:**

A cross-sectional study was carried out on 327 children aged 12-36 months. The data collected included anthropometric measurement, serum hemoglobin (Hb), and vitamin D concentration. Blood analysis was done at the Center Laboratory of Hungyen Obstetrics and Pediatrics Hospital. Underweight, stunted, and wasted children were classified based on z-scores cut-off less than -2 SD of weight for age (WAZ), height for age (HAZ), and weight for height (WHZ), respectively. Overweight and obese children were defined if WHZ was more than + 2SD. Anemic child was applied when Hb concentration was less than 110 g/L while vitamin D deficiency was termed for level less than 20 ng/L.

**Results:**

The prevalence of underweight, stunted, wasted, and overweight/obese children was 7.6%, 23.5%, 6.7%, and 1.2%, respectively. The prevalence of anemia and vitamin D deficiency was 33.3% and 47.7%, respectively. Malnutrition, anemia, and vitamin D deficiency were not statistically different by sex. Malnutrition and vitamin D deficiency were not statistically different by age group but anemia by age groups was significantly different.

**Conclusions:**

Stunting is still prevalent in children aged 12-36 months in Kimdong. Moreover, anemia and vitamin D deficiency also affected children in this area. Some interventions should be conducted to improve the nutritional status of children in Kimdong district.

## 1. Introduction

Malnutrition, very common in children less than 5 years old, creates a major public health problem in developing countries including Vietnam. It is closed in a vicious cycle that can increase the morbidity and mortality rate and poor health in children. Malnutrition can influence physiopsychological development that leads to lower performance and productivity when children grow up [1].

The most common form of malnutrition is stunting that affected 156 million children in current worldwide scale [2]. According to Da Silva ICM et al., global stunting prevalence has been nearly halved between 1990 and 2016, but it is still not clear whether this decline has happened in poor rural population within low and middle income countries [3].

According to the study of Screeramareddy CT et al. (2014) the incidence of stunting, wasting, and underweight of children less than 5 years old in Nepal was remarkably high. They were 41.6%, 11.5%, and 30.1%, respectively [4].

In Vietnam, although many efforts of the state government and local governments have been made to improve nutritional status of children less than 5 years old, the results obtained were not good enough to change, overall, children's nutritional status. A study done by Tran, N.T.N in Gialoc, Haiduong (2017), a neighboring province of Hungyen, showed a significantly high incidence of stunting (25,9%) [5].

According to the Ministry of Health of Vietnam, Hungyen is among 22 leading provinces where the malnutrition level is very high [6]. Kimdong is one of the poorest among 9 districts of Hungyen. Therefore, Kimdong was chosen by Hungyen Obstetrics and Pediatrics Hospital to conduct this study on the nutritional status in children aged 12 to 36 months. Results of the study will help researchers implement some nutritional interventions to improve the nutritional status for children in this area.

## 2. Subjects and Methods

### 2.1. Research Design and Subjects

The study was conducted using a cross-sectional design. Three hundred and twenty-seven children from 12 to 36 months of age from Kimdong district, Hungyen Province in North Vietnam, were involved in this study, in October 2017. The sample size was calculated based on the national stunting prevalence among children in Vietnam [6]. Stunting is always highest among children with malnutrition. A multistage sampling process was chosen. Five communes (Ngocthanh, Vinhxa, Hungan, Hiepcuong, and Dongthinh) were selected randomly among 22 communes of Kimdong district while study subjects were chosen by systematic sampling method based on the total of 1.160 children aged 12-36 months in those five communes to reach the final number of 327 children.

#### 2.1.1. Inclusion and Exclusion Criteria

Eligible children were aged 12-36 months with their parents.

Children with congenital diseases or children who had been taking some polyvitamins, including iron or vitamin D, were excluded.

Parents refused to participate in this study or have lived in Kimdong district less than 6 months.

#### 2.1.2. Data Collection Procedures

The data was collected at each commune health station, including general information, anthropometric measurement, and venous blood sample as below.

*General Data*. Demographic data was collected by interviewing the parents. The age of children was calculated based on WHO definition [7].

*Anthropometric Measurement*. The weight: Automatic weight scale (SECA-769) with a precision of 10 g was used to measure the body weight of the participants. The scale was calibrated at the time of each measurement.

The length/height: For children under 2 years of age, their length was measured in recumbent position using wood scale (set-square tool) with a precision of 0.1 cm. The height was measured for children over 2 years old by using Microtoise height scale with a precision of 0.1 cm.

Blood sampling: Blood was taken to assess Hb and vitamin D level. Three cubic milliliters were taken intravenously, in the morning between 7 am and 8 am, before breakfast.

#### 2.1.3. Definitions

Classification for underweight, stunting, and wasting children and their severity was based on WHO criteria, with the cut-off below -2 SD [7].

Underweight was defined as z-score of weight for age (WAZ) less than -2 SD. When WAZ was -2 SD-<-3 SD, -3 SD-<-4 SD, and <- 4 SD that corresponded to the first (mild), second (moderate), and third degree (severe), respectively.

Stunting was defined as z-score of height for age less than -2 SD. When HAZ was -2 SD-<-3 SD, -3 SD-<-4 SD, and <- 4 SD that corresponded to the first, second, and third degree, respectively [8].

Wasting was defined as z-score of weight for height (WHZ) less than -2 SD. When WHZ was -2 SD-<-3 SD, -3 SD-<-4 SD, and <- 4 SD that corresponded to the first, second, and third degree, respectively.

Overweight and obese children were defined as WHZ more than + 2 SD.

Anemia was defined as serum hemoglobin concentration less than 110g/L [8]. Mild, moderate, and severe anemia were defined as Hb level 100-109 g/L, 70-99 g/L, and <70 g/L, respectively [9, 10]. Vitamin D deficiency was defined when serum vitamin D concentration was less than 20 ng/mL and vitamin D insufficiency as serum vitamin D concentration from 20 to 30 ng/mL [11].

### 2.2. Data Analysis

All anthropometric z-scores parameters were calculated by using WHO Anthro software 2006 [7]. Continuous data was described by mean and standard deviation. To compare continuous data, 2 percentages, the Student t-test, and the Chi-square were analyzed by SPSS software version 22.0 (SPSS Inc., Chicago, IL, USA). P-value less than 0.05 was used to define statistical significance.

Total serum vitamin D concentration was assessed by enzyme-linked immunosorbent assay (ELISA) technique using Architeqch-i100SR-Abbott Link.

Hb level was measured by spectrophotometric method on Cell Dyn 3700 machine.

Blood analysis was done at the Obstetrics and Pediatrics Hospital Center Laboratory, Hungyen Province.

### 2.3. Ethical Considerations

Study protocol was accepted by Hungyen Obstetrics and Pediatrics Hospital (No 109/QD-HDKH) and approved by Haiphong University of Medicine and Pharmacy Institutional Review Board (No. 35/HDDD). The parents or legal guardians of these children gave informed consent after full explanation of the objectives of the study was provided. Each participant in the final sample was identified by assigning a number.

## 3. Results

### 3.1. Participants' Demographic Characteristics

The mean age was 23.93 ± 7.04 months and more than half of the participants were boys. The children aged 12-<24 months were 51.07% and the children aged 24-<36 months were 48.93%.

The mean weight was 11.16 ± 1.85 kg and the mean height was 83.65 ± 6.85 cm. The mean WAZ, HAZ, and WHZ values were -0.49 ± 1.12 z-score, -0.82 ± 1.33 z-score, and -0.14 ± 1.21 z-score, respectively.

The mean of Hb level was 112.84 ± 12.32 g/L and of vitamin D level was 32.52 ± 10.59 ng/mL (Table 1).

### 3.2. The Distribution of WAZ, HAZ, WHZ, Hb Level, and Total Vitamin D Concentration according to Sex and Age Group

#### 3.2.1. Anthropometric and Biochemical Assessments among Study Subjects

The mean of WAZ for boys was -0.92 ± 1.16 and for girls was -0.73 ± 1.06. The difference was not statistically significant between genders. The mean HAZ for boys was -0.59 ± 1.12, significantly lower compared with that of girls, -0.37 ± 1.06 z-scores with p = 0.055. The difference in the mean WHZ for boys and girls was not statistically significant.

There was not statistically significant difference between boys and girls in terms of Hb and vitamin D level (p> 0.05) (Table 2).

The mean WAZ for the 12-<24 months group was -0.29 ± 1.12 z-score, significantly higher than that of the 24-<36 months group (-0.99 ± 0.00) (p<0.01). The mean of HAZ for the 12-<24 months group was -0.73 ± 1.17 z-score, showing tendency to be lower than that of the 24-36-month group (-0.94 ± 1.05 z-score) (p>0.05). The mean of WHZ for the 12-<24 months group was -0.84 ± 1.19 z-score, significantly higher than that of the 24-36-month group (-0.42 ± 1.34 z-score) (p<0.,01). The mean of WAZ and WHZ indicated that children in the 24-36-month group were more likely to be underweight and children in the 12-<24 months group were more likely to be wasted (Table 3).

Children in the 12-<24 months group had higher risk of anemia than that of the 24-36-month group (p<0.001). The 24-36-month group was more likely to be vitamin D deficient than the 12-<24 months group but the difference was not statistically significant with p>0.05 (Table 3).

### 3.3. The Nutritional Status of the Study Subjects

The prevalence of underweight, stunting, and wasting was 7.6%, 23.5%, and 6.7%, respectively. Most of these undernourished children were mild cases. No severe case was found in this study. The rate of overweight children was 1.2%. Anemic children accounted for 33.3% of the studied subjects. Vitamin D deficient children accounted for 47.7%. Almost all cases were mild (Table 4).

There was not statistically significant difference between girls and boys in terms of percentage of undernourished, anemic, and vitamin D deficient children (p>0.05) (Table 5).

We did not find any statistically significant difference by age groups in terms of the prevalence of underweight, stunting, wasting, and vitamin D deficiency in participants. But there was statistically significant difference by age groups of anemia prevalence (Table 6).

## 4. Discussion

### 4.1. Prevalence of Malnutrition

A total of 327 children aged 12-36 months in Kimdong, Hungyen, Vietnam, were investigated, in which boys were 52.9% and girls were 47.1%. The proportion of 12-<24 months was 51.1% while the 24-36-month group was 48.9%. The mean age for both sexes was 23.93 ± 7.04 months. The mean WAZ, HAZ, and WHZ for both sexes were -0.49 ± 1.12, -0.82 ± 1.33, and -0.14 ± 1.21, respectively. The mean height and weight z-score for both sexes were 11.16 ± 1.85 cm and 83.65 ± 6.85 kg, respectively.

Stunting had the highest prevalence among the factors studied, representing 23,5%, in which 9.88% were mild cases. Underweight accounted for 7.5%, of which 7.34% were mild. Wasted children accounted for 6.7%, of which 6.42% were mild ones. No severe cases were reported among undernourished children. No difference was found based on age and gender.

The stunting prevalence in the study (23,5%) was slightly lower than the national level reported (24.6%), whereas our underweight prevalence was much lower than the national documented level in 2015 (14.1%) [12]. Compared with results from Tran, NTN [5] in Gialoc, Haiduong Province, a rural district having the same socioeconomic conditions as our study, the stunting and underweight prevalence were significantly lower (23.5% versus 25.9% and 7.5% versus 11.8%, respectively), while the wasting prevalence was higher (6.7% versus 3.0%). The underweight and stunting prevalence found in our study were a bit lower compared with national data and Tran, NTN's result. That can be explained by the influence of positive socioeconomic development over time in Vietnam. Although the stunting prevalence has been decreasing over the years, it is still high and should be addressed by leaders in different levels of health services in Kimdong.

According to results from studies conducted in different regions and different times in Vietnam, we noted that there was a big difference in malnutrition prevalence among studies. Do, H.T, found that 27.8% of children under 5 years old in 2 coastal communes of Tienlang, Haiphong, in 2011 were stunted [6]. Another study done by Khong CM in a rural district of Haiphong in 2012 found that 21.9% of participants suffered from stunting malnutrition [13]. Stunting malnutrition in children under 5 years old in an urban county of Haiphong in 2013 was studied by Le, M.T, and the result of the study showed that 20.7% of participants suffered from stunting malnutrition [14]. These results revealed that stunting malnutrition prevalence in children under 5 years old in coastal and rural areas of Haiphong was higher than that of urban areas. Similarly, the research done by Tran, T.D, in a rural district of Hungyen in children under 5 years old to estimate the malnutrition prevalence showed that 27.5% of studied subjects were stunted [15].

Recent research investigated children less than 5 years old in Kimthanh, Haiduong 2018, a region being urbanized. The results showed there was a low malnutrition prevalence: underweight was 7.6%, stunting was 11.8%, and wasting was 1.6% [16].

All the studies mentioned above showed there are differences in malnutrition prevalence between rural/coastal and urban areas of Vietnam.

The same studies in children under five years old done in rural areas of Pakistan [17], Iraq [18], Sudan [19], Tanzania [20, 21], Bangladesh [22], Cambodia [23], and mountainous areas of Nepal [24, 25] and Haiti [26] showed as high prevalence of malnutrition as our results. The stunting prevalence ranged from 14.4% to 56.0%, the underweight prevalence ranged from 15.5% to 33.3%, and the wasting prevalence ranged from 4.5% to 21.0%.

While studying malnutrition in children under five years old in 67 countries with moderate and low income in 1993, Da Silva ICM et al. [3] found that, on average, 53.7% of children under five in low income countries were stunted, and in countries with moderate income this prevalence was 48.2%.

We also found that malnutrition was mild and moderate while studies done in other developing countries showed high prevalence of moderate and severe malnutrition [24, 27].

At present, stunting is of concern to many developing countries, including Vietnam. In world perspective on the epidemiology of stunting between 1990 and 2015, Campisi SC et al. [2] found that, in 2015, there were 98.5 million fewer stunted children less than 5 years old than in 1990. In East Asia and Pacific and South Asia, the stunting prevalence decreased by 24.8% and 25%, respectively. Minimal declines were observed in Latin America and the Caribbean at 12.6%, in the Middle East and North Africa at 12.9%, and in Sub-Saharan Africa at 13.4%, but, in Sub-Saharan Africa, the number of stunted children increased by 12.4 million between 1990 and 2015. These results showed that stunting is of broad international interest and many efforts have been made to reduce prevalence of the stunting.

The malnutrition prevalence difference between our finding and other studies done in developing countries in the world was because our research subjects lived in areas with different levels of socioeconomic conditions and were vulnerable to different levels of risk factors. Compared with results of similar researches done in Vietnam, our malnutrition prevalence was much lower than average national data. Malnutrition in Kimdong was still high and further improvement is needed.

### 4.2. Overweight and Obesity Prevalence

In our study, there were only 4 overweight/obese children, accounting for 1.2%. Our result was very similar to that of Tran, T.N.T in Gialoc, Haiduong, in 2017 (1.4%).

In urban areas of Vietnam, children currently are more likely to be obese. According to a longitudinal study of a cohort of 2.677 children aged 3 to 6 years old in Hanoi of Do, L.M, et al. [28], in 2013-2016, the overall estimated prevalence of overweight increased from 9.1% to 16.7%. This prevalence was higher than that of Kimdong (1.4%).

In 2012, the overweight and obesity prevalence in Iraqi children who were less than 5 years old was 7.2% [18], considerably higher than our finding (1.4%).

Rachmi CN et al. [29] in Indonesia found that malnutrition of all kinds was very common in children aged 2-4.9 years in 13 out of 27 provinces and overweight/obesity increased from 10.3% to 16.5%.

Although overweight and obesity prevalence in Kimdong was low, it indicated a beginning tendency of overweight and obesity in rural areas of Vietnam. Like many other developing countries [30–32], Vietnam is experiencing the double burden of undernutrition and excess body weight. However, Kimdong district was not really touched by the double burden of malnutrition that is very common in many other developing countries [29].

### 4.3. Anemia Prevalence

The prevalence of anemia in Kimdong was 33.3% and the prevalence in the 12-<24 months group was 44.3% and the prevalence in the 24-36 months was 21.9%. Younger children were more likely to be anemic than older ones (p<0.001). Anemia prevalence was not significantly different according to sexes (boys 35.26% and girls 31.17%; p>0.05). From this prevalence of anemia for both sexes, we found that about 1/3 of studied children suffered from anemia. 30.27% and 3.06% of children were mild or moderately anemic, respectively. In this study, we only focused on determining the prevalence of anemia but not on its risk factors.

In 2009, Nguyen, P.H, et al. [33] showed that 60% of children less than 2 years old in her study were anemic. This prevalence was much higher than that of Kimdong.

Compared to research done in other developing countries in the world, anemia prevalence in Kimdong was quite low. For example, Tiku YS et al. (2016) [34] carried a study in children aged 6-50 months in Duggina Fanigo District, Southern Ethiopia, and 51.4% of children there were anemic. In another study, Kejo D et al. [35] showed that 84.6% of 436 children aged 6-59 months in Arusha District, Tanzania, were found to be anemic. According to a study of Ncogo P et al. (2013) [36] in urban and rural settings from Basa District, Guinea, 1,421 children were screened for anemia, of whom more than 85% were anemic. When conducting a cohort study including 376 households having children aged 6 to 59 months, in which, 68.5% of children were 12-23 months, in Namutumba district, Uganda, Kuziga F et al. [37] recognized that the prevalence of anemia was 58.5%, much higher than our results (33.3%).

In terms of anemic severity, the number of children with mild cases was the highest. Ncogo P et al. [36] indicated that, among 85% anemic children, 24%, 6%, and 9% were mild, moderate, and severe, respectively.

The differences in anemia prevalence found in our study and published data can be explained by the differences of the age of participating children [37], the time point in which studies were carried out, the research area (urban or rural settings) [36], and the research subjects being exposed or not exposed to risk factors for anemia [35, 36]. Another factor that might contribute to the difference was the blood sampling and analysis method. For example, blood samples from the fingertip or from the vein, blood analysis by portable equipment, or equipment in the laboratory.

According to Kassebaum NJ et al. [38], global anemic prevalence from 1990 to 2010 in 187 countries, both sexes, and 20 age groups was 32.9%. The burden of anemia was highest in geographic regions such as South Asia and Central, West, and East Sub-Saharan Africa and in children under age of 5.

### 4.4. Vitamin D Deficiency Prevalence

Our research indicated that the prevalence of vitamin D deficiency was 47.7% of which 45.56% were insufficiency and 2.14% were real deficiency. The vitamin D deficiency differed statistically in age groups but not in sexes. In our research setting, vitamin D insufficiency was very prevalent.

Data has shown that the prevalence of vitamin D insufficiency in Kimdong was relatively similar to that of Tran, NTN [5] (45.56% versus 46.4%, respectively), but the prevalence of vitamin D deficiency was lower than that of Tran, N.T.N (2.14% versus 49.0%) [5]. The difference in vitamin D deficiency can be explained by the different time points at which study was carried out and positive socioeconomic development (which can reduce vitamin D deficiency prevalence in children in general). According to the Nutrition Institute of Vietnam 2015, the prevalence of vitamin D deficiency in children of urban and rural areas was 62.1% and 53.7%, respectively. Compared with these data, our prevalence was much lower (47.7%).The study conducted by Vu, H.T.T., et al. in children aged from 1 to 6 months in 2012 in Hanoi showed that 64.3% of surveyed children had vitamin D concentration less than 75nmol/l [39]. This prevalence was similar to that of the national one in 2015 and higher than that of our study in 2017.

Vitamin D deficiency was studied in many countries in the world [32, 38–43]. The study done by Mandlik R et al. [44] in Iran in school-children aged 6-12 years in rural and semirural areas showed high prevalence of vitamin D insufficiency (71.0%). Ahmed P et al. [45] found that 50% of children aged 2-60 months had vitamin D level less than 50 nmol/L. It was shown that vitamin D deficiency is not limited to particularly geographic regions but it is also related to countries with tropical climate conditions. Vitamin D deficiency may be related to another mechanism, an issue for further study.

### 4.5. Some Strengths and Weaknesses

The study had some strengths such as the fact that it was well designed and blood test for anemia and serum vitamin D concentration were done. However, it had some weaknesses. This study focused on narrow age group of 12 to 36 months among children under five years old. Results from this study were used to implement some interventions in children under five years old in the studied setting that was able to create some bias. Furthermore, the study was done in October of year 2017 when it became cold in North Vietnam and the mean sunlight hour a day reduced. That probably made the vitamin D deficiency prevalence in children increase.

## 5. Conclusions

The incidence of underweight, stunting, and wasting in Kimdong districts, Haiduong Province, Vietnam, was 7.6%, 23.5%, and 6.7%, respectively. Regarding malnutrition, mild and moderate forms were the most frequently encountered. The incidence of overweight and obesity was 1.2%. Anemia and vitamin D deficiency were found in 33.3% and 47.7% of subjects, respectively.

## 6. Recommendations

(i) The children nutritional status should be explained to their parents as well as local health service leaders so they can intervene to address that situation.

(ii) Some community-based interventions should be implemented to improve children's nutritional status, particularly to address stunting, anemia, and vitamin D deficiency.

## Figures and Tables

**Table 1:** Demographic characteristics of research subjects (n=327).

Characteristics	n	%	Mean ± SD
Number of participants	327		

Sex (boy)	173	52.9	

Mean age (months)	327		23.93 ± 7.04

Age group (months)			

12-<24	167	51.07	

24-<36	160	48.93	

Weight (kg)	327		11.16 ± 1.85

Height (cm)	327		83.65 ± 6.85

WAZ	327		-0.49 ± 1.12

HAZ	327		-0.82 ± 1.33

WHZ	327		-0.14 ± 1.21

Hb concentration (g/L)	327		112.84 ± 12.32

Vitamin D level (ng/mL)	327		32.52 ± 10.59

**Table 2:** The distribution of subjects' WAZ, HAZ, WHZ, Hb level, and total vitamin D concentration according to sex.

Characteristics	Number	Mean ± SD	p-value (2-tailed)
WAZ^*∗*^			

Boy	173	-0.92 ± 1.16	>0.05
Girl	154	-0.73 ± 1.06	

HAZ^*∗*^			

Boy	173	-0.59 ± 1.12	=0.055
Girl	154	-0.37 ± 1.06	

WHZ^*∗*^			

Boy	173	-0.92 ± 1.16	>0.05
Girl	154	-0.73 ± 1.05	

Hb (g/L)^*∗*^			

Boy	173	112.45 ± 11.03	>0.05
Girl	154	113.28 ± 10.08	

Vitamin D (ng/mL)^*∗*^			

Boy	173	33.65 ± 15.23	>0.05
Girl	154	31.25 ± 7.72	

^*∗*^Student t-test.

**Table 3:** The distribution of research subjects' WAZ, HAZ, WHZ, Hb level, and vitamin D concentration according to age group.

Characteristics	n	Mean ± SD	p-value (2-tailed)
WAZ^*∗*^			

12-<24 months	167	-0.29 ± 1.12	<0.01
24-36 months	160	-0.99 ± 0.00	

HAZ^**∗**^			

12-<24 months	167	-0.73 ± 1.17	>0.05
24-36 months	160	-0.94 ± 1.05	

WHZ^*∗*^			

12-<24 months	167	-0.84 ± 1.19	<0.01
24-36 months	160	-0.42 ± 1.34	

Hb (g/L)^*∗*^			

12-<24 months	167	110.49 ± 11.03	<0.001
24-36 months	160	115.30 ± 9.37	

Vitamin D (ng/mL)^*∗*^			

12-<24 months	167	32.26 ± 9.13	>0.05
24-36 months	160	32.79 ± 14.97	

^*∗*^Student t-test.

**Table 4:** The prevalence and the severity of malnutrition, anemia, and vitamin D deficiencies among the study subjects.

Nutritional status	Investigated subjects	Number of cases	Percentage (%)
*Underweight*	327	25	*7,6*

Mild		24	**7.34**

Average		1	**0.31**

Severe		0	**0.00**

*Stunting*	327	77	*23.5*

Mild		65	**19.88**

Average		12	**3.67**

Severe		0	**0.00**

*Wasting*	327	22	*6.7*

Mild		21	**6.42**

Average		1	**0.31**

Severe		0	**0.00**

*Anemia*	327	109	*33.3*

Mild		99	**30.27**

Moderate		10	**3.06**

Severe		0	**0.00**

*Vitamin D Deficiency*	327	156	47.7

Insufficiency		149	**45.56**

Deficiency		7	**2.14**

*Overweight/obesity*	327	4	*1.2*

**Table 5:** The prevalence of malnutrition, anemia, and vitamin D deficiency by gender.

Prevalence/Sex	Investigated subjects	Number of cases	Percentage (%)	p-value (2-sided)
Underweight (n=25)^*∗*^				

Boy	173	15	8.67	>0.05
Girl	154	10	6.49	

Stunting (n=77)^*∗*^				

Boy	173	45	26.01	>0.05
Girl	154	32	20.78	

Wasting (n=22)^*∗*^				

Boy	173	13	7.51	>0.05
Girl	154	9	5.84	

Anemia (n=109)^*∗*^				

Boy	173	61	35.26	>0.05
Girl	154	48	31.17	

Vitamin D deficiency (n=171)^*∗*^				

Boy	173	81	46.80	>0.05
Girl	154	75	48.70	

^*∗*^Chi-square test.

**Table 6:** Prevalence of nutritional status by age group.

Prevalence/Age group	Investigated subjects	Number of cases	Percentage (%)	p-value (2-sided)
Underweight (n=25)^*∗*^				

12-<24 months	167	11	6.58	>0.05
24-36 months	160	14	8.75	

Stunting (n=77)^*∗*^				

12-<24 months	167	42	25.15	=0.05
24-36 months	160	35	21.87	

Wasting (n=22)^*∗*^				

12-<24 months	167	7	4.19	>0.05
24-36 months	160	15	9.37	

Anemia (n=109)^*∗*^				

12-<24 months	167	74	44.31	<0.001
24-36 months	160	35	21.87	

*Vitamin D* deficiency (n=171)^*∗*^				

12-<24 months	167	71	42.50	=0.055
24-36 months	160	85	53.10	

^*∗*^Chi-square test.

## Data Availability

The EXCEL/SPSS data used to support the findings of this study are available from the corresponding author upon request.

## Abbreviations

Hb: Hemoglobin
SD: Standard deviation
WAZ: Weight for age z-score
HAZ: Height for age z-score
WHZ: Weight for height z-score.
